# HIV Increases the Risk of Cigarette Smoke-Induced Emphysema Through MMP-9

**DOI:** 10.1097/QAI.0000000000003125

**Published:** 2022-11-04

**Authors:** Bashar S. Staitieh, Simran Malik, Sara C. Auld, Gregory W. Wigger, Xian Fan, Andrew T. Roth, Tanima Chatterjee, Itika Arora, S. Vamsee Raju, Sonya Heath, Saurabh Aggrawal

**Affiliations:** a Department of Medicine, Division of Pulmonary, Allergy, Critical Care & Sleep Medicine, Emory University School of Medicine, Atlanta, GA;; b Department of Medicine, Division of Pulmonary and Critical Care Medicine, Washington University School of Medicine in St. Louis, St. Louis, MO;; c Department of Epidemiology, Rollins School of Public Health, Emory University, Atlanta, GA;; d Department of Anesthesiology and Perioperative Medicine, University of Alabama at Birmingham, Birmingham, AL;; e Division of Pulmonary, Allergy, and Critical Care Medicine, University of Alabama at Birmingham, Birmingham, AL; and; f Department of Medicine, Division of Infectious Disease, University of Alabama at Birmingham, Birmingham, AL.

**Keywords:** emphysema, HIV, smoking, metalloproteinase, Tat

## Abstract

HIV is associated with an increased risk for emphysema. Matrix metalloproteinase 9 (MMP-9) is a lung tissue remodeling enzyme associated with emphysema. We previously found MMP-9 activity increases with increases in oxidative stress and that HIV increases alveolar oxidative stress. We hypothesized that HIV proteins would increase the risk of cigarette smoke-induced emphysema due to MMP-9.

**Methods:**

HIV-1 transgenic rats and wild-type littermates were exposed to cigarette smoke or sham for 8 weeks. Lung compliance and histology were assessed. Bronchoalveolar lavage (BAL), primary alveolar macrophages (AM), and serum samples were obtained. A rat alveolar macrophage cell line was exposed to the HIV protein Tat, and MMP-9 levels were assessed by Western immunoblotting. MMP-9 protein expression and activity were assessed in AM from the HIV rat model by ELISA and cytoimmunofluoresence, respectively. Serum from human subjects with and without HIV and tobacco dependence was assessed for MMP-9 levels.

**Results:**

MMP-9 expression was significantly increased in rat alveolar macrophages after Tat exposure. HIV-1 transgenic rats developed emphysema while wild-type littermates did not. MMP-9 expression was also increased in the serum, BAL, and AM of HIV-1 transgenic rats after exposure to cigarette smoke compared with wild-type rats. In parallel, serum samples from HIV+ smokers had higher levels of MMP-9 than subjects without HIV and those who did not smoke.

**Conclusion:**

The combination of HIV and cigarette smoke increases MMP-9 expression in experimental rat HIV models and human subjects. HIV and cigarette smoke both induce alveolar oxidative stress and thereby increase MMP-9 activity.

## INTRODUCTION

Globally, nearly 40 million people are living with HIV, with 1.7 million new infections in 2019 alone.^1^ In the United States, more than half of those with HIV are smokers, compared with 20% of the general population.^2–4^ In addition to the higher risk of all-cause mortality in this population,^5,6^ smokers with HIV are more likely to have a poor response to antiretroviral therapy (ART) and are vulnerable to a host of serious HIV-related complications.^5–8^ Although ART has significantly reduced the rates of opportunistic pulmonary infections that were historically the most common cause of death in people with HIV,^9^ it has had substantially less impact on reducing the rates of noninfectious pulmonary complications.^10^ To date, a number of cohort studies have demonstrated increased rates of noninfectious pulmonary complications in people with HIV.^11^ The largest such study analyzed data from the Veterans Aging Cohort and found that people with HIV had a 25% greater incidence of emphysema than people without HIV, even after adjustment for risk factors such as smoking.^12^

Unfortunately, it remains unknown whether the biological drivers of HIV-associated emphysema are different than those for people without HIV. Emphysema is a complex disorder that results from the confluence of multiple factors, including an imbalance between proteases and antiproteases, oxidative stress, and chronic inflammation. Several hypotheses have been advanced in the literature to explain why people with HIV develop emphysema at higher rates, including their high rates of tobacco use and their more frequent development of coinfections that could lead to permanent structural damage (eg, *Pneumocystis* pneumonia and tuberculosis). However, our understanding of the mechanisms underlying HIV-associated emphysema remains relatively superficial.^13,14^ A better understanding of how emphysema occurs in HIV will help us to develop therapies specifically targeted to HIV-associated emphysema, rather than our current strategy of treating it as we would COPD of any other etiology.

In our efforts to elucidate a potential mechanism for HIV-associated emphysema, we turned to the matrix metalloproteinases (MMPs), a collection of degradative enzymes that can remodel lung tissue after injury or destroy it when activated overexuberantly. We previously determined that MMP-9 activity increases in the setting of oxidative stress,^15^ and our group and others have previously shown that HIV leads to increases in oxidative stress in the alveolar space.^16^ We therefore hypothesized that cigarette smoking increases the risk of emphysema in HIV because of increased expression and activity of MMP-9. We tested this hypothesis with a series of experiments using a well-established rat model of chronic HIV^16^ alongside cell-based assays and human samples from smokers and nonsmokers with and without HIV.

## METHODS

### Human Subjects Cohort

Study participants were consecutively recruited from the 1917 HIV clinic at the University of Alabama (UAB) as part of an ongoing longitudinal cohort study (UAB IRB-300003918). Participants were divided into 4 groups: (1) 20 nonsmokers without HIV, (2) 20 smokers without HIV, (3) 20 nonsmokers with HIV, and (4) 19 smokers with HIV. All people with HIV were on ART, and all smokers had a minimum 5-year history of cigarette smoking. Participants with an active bacterial or non-HIV viral infection, on the basis of patient interviews and medical record review, were not eligible for this substudy. Blood was collected at the 1917 clinic through venipuncture and processed to obtain plasma^17,18^ within 2–4 hours. Plasma MMP-9 and RAGE levels were measured using the Quantikine ELISA Human MMP-9 and RAGE Immunoassay (R & D Systems, Minneapolis, MN).

### HIV-1 Transgenic Rat Model

Adult Fischer 344/NHsd HIV-1 Transgenic (HIV Tg) rats and their wild-type littermate controls (Fischer 344) were used for these experiments. This rat model lacks the *gag* and *pol* genes necessary for functional virion formation, but otherwise produces the full spectrum of HIV-related proteins, including Tat, Rev, Nef, and gp120.^19^ All procedures were approved by the Institutional Animal Care and Use Committee at Emory University or the University of Alabama-Birmingham.

### Cigarette Smoke Exposure

Adult male HIV transgenic rats (4–5 months old) and their wild-type littermate controls (Fischer 344) were exposed to either cigarette smoke or sham. After a brief period of acclimatization and training, rats were exposed to cigarette smoke (CS) as previously described^20^ using standardized 1R6F research cigarettes (University of Kentucky, Lexington, KY) for 120 minutes once daily, 5 days a week, for 2 months. Control male rats were placed in identical chambers for the same duration without cigarette smoke.

### Assessment of Respiratory Mechanics

Pressure–volume (PV) curves were assessed in anesthetized rats as previously described using the FX-1 module of the FlexiVent (SCIREQ).^21^ Lung compliance (C, Δ*V*/Δ*P*) was derived from the PV curves on the deflation (upper) limb between lung pressures of 7 and 11.4 cm H_2_O under closed-chest conditions to assess the intrinsic elastic properties of the respiratory system (ie, lung + chest wall) at rest.

### Lung Histology

Rat lung tissues were removed and fixed in 70% alcoholic formalin for 24 hours and dehydrated in 70% ethanol before embedding in paraffin for assessment of mean linear intercept (L_m_) as previously described.^21^ In brief, L_m_ was measured by dividing the total length of lines drawn across the lung fields by the number of intercepts with alveolar septum at ×20 magnification. Alveolar number was determined by the number of measurements made for the L_m_.^21^

### Bronchoalveolar Lavage & Cell Culture Treatments

Primary alveolar macrophages (AM) were obtained by bronchoalveolar lavage from ∼10-month-old HIV Tg rats and their WT littermate controls. A total cell count was completed. Total protein levels were measured using a BCA protein assay (Thermo Fischer Scientific, Waltham, MA). Cells were plated for 2 hours before washing to allow for removal of nonadherent cells and retention of a relatively purified macrophage population (>95%) as previously described.^22^ Cells were then treated ± 5 nM CAS 1177749-58-4 (MMP-9 inhibitor, Calbiochem, San Diego, CA) for 24 hours. In parallel, alveolar macrophages from NR8383 cells, a rat alveolar macrophage cell line (ATCC, Manassas, VA) were plated at a density of 1 M cells/well on a 6-well plate and treated with 10 ng/mL Tat × 48 hours.

### Western Blotting

Total proteins were isolated from alveolar macrophages using Laemmli sample buffer (Bio-Rad Laboratories, Hercules, CA) before protein electrophoresis as previously described.^23^ After gel transfer, membranes were incubated with anti-MMP-9 (Abcam, Waltham, MA) and anti-GAPDH as a loading control overnight before incubation with an antirabbit secondary antibody. Immunoreactive bands were captured with ChemiDoc XRS system (Bio-Rad Laboratories, Hercules, CA) after application of an ECL substrate (GE Healthcare Life Sciences, Pittsburgh, PA). Relevant bands were identified by molecular weight and quantified using the ratio of target to loading-control densitometry.

### Cytoimmunofluorescence

Primary rat alveolar macrophages were isolated and plated on a 16-well chamber slide as previously described^24^ before treatment with or without 5 nM of MMP-9 inhibitor. Wells were then fixed with 4% paraformaldehyde and incubated with polyclonal rabbit antibodies to MMP-9 (Abcam) or receptor for advanced glycation end (RAGE) antibody (Abcam, Waltham, MA) overnight at 4 °C before incubation with an antirabbit Alexa Fluor 488 secondary antibody (Jackson ImmunoResearch, West Grove, PA) and DAPI (Vector Laboratories, Burlingame, CA) for nuclear staining. Images of 80–100 alveolar macrophages/experimental group were acquired with an Olympus fluorescent microscope (Center Valley, PA). Random fields were selected from each slide and at least 5 images were acquired per sample for a total of 80–100 cells/condition. ImageJ (NIH, Bethesda, MD) was used to perform automated background correction and to process the images into a binary image map to allow for automated cell border acquisition and assessment of mean fluorescence intensity.

### MMP-9 ELISA

MMP-9 levels were measured in plasma and BAL cell lysate from HIV transgenic rats and their littermate controls using the Quantikine ELISA Rat Total MMP-9 Immunoassay (Cat No.-RMP900, R&D Systems, Minneapolis, MN) according to manufacturer's guidelines.

### Statistical Analyses

Student *t* test was used for single comparisons. In studies with more than 2 groups, statistical significance was calculated using one-way ANOVA followed by Tukey–Kramer post hoc tests to detect differences between individual groups (GraphPad Prism version 5, San Diego, CA). Data are presented as mean ± SEM. Significance was accepted at *P* < 0.05.

## RESULTS

### Human Subject Recruitment

Sociodemographic characteristics did not differ significantly between study groups (average age range 42–48 years; race distribution 44%–58% African American; 20%–25% female sex) (Table 1). As with the patient population of the 1917 HIV clinic, smokers with HIV enrolled into this study were predominantly men. The average current and nadir CD4^+^ cell count and average current viral load (VL) were not different between smokers and nonsmokers with HIV. COPD and hypertension rates were higher in subjects with HIV. All subjects with HIV were on antiretroviral therapy.

**TABLE 1.** Baseline Characteristics

Group	Nonsmokers Without HIV (N = 20)	Smokers Without HIV (N = 20)	Nonsmokers With HIV (N = 20)	Smokers With HIV (N = 19)
Mean age (SD)	42 (9.4)	45 (6.7)	48 (9.8)	47 (10.9)
Female, n (%)	4 (20)	4 (20)	5 (25)	5 (25)
African American, n (%)	52	56	44	58
Mean CD4^+^ (SD)			855.7 (318)	872.9 (389.2)
Mean nadir CD4^+^ (SD)			351.8 (351.8)	355.3 (191.1)
Mean viral load (SD)			Undetectable	Undetectable
COPD (%)			5	25
Hypertension (%)	3.7	21.4	69.5	38
Antiretroviral therapy (%)			100	100

SD, standard deviation.

### HIV-1 Transgene Expression Causes Emphysema in Rat Lungs

**FIGURE 1.**
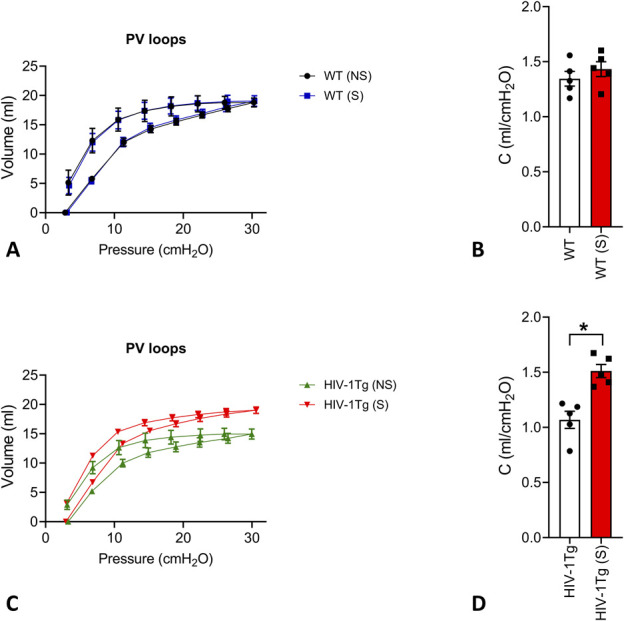
Cigarette smoke exposure does not alter the lung compliance of wild-type rats but significantly increases lung compliance in HIV transgenic rats. Four to 5-month-old male HIV Tg rats and their littermate controls were exposed to cigarette smoke (CS) or sham for 8 weeks prior assessment of lung mechanics under closed-chest conditions. CS did not appreciably alter the mechanics of the WT rats (A–B) but caused a significant increase in the compliance of HIV Tg rats (C–D). All animals were males (n = 5–6). Individual values and means ± SEM. *P < 0.05 vs. groups at the end of individual lines.

**FIGURE 2.**
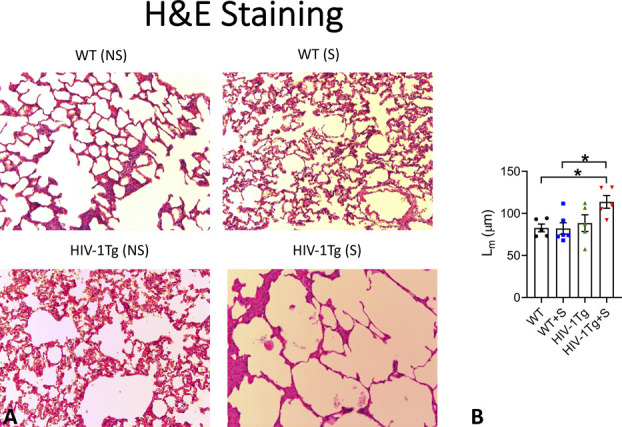
Cigarette smoke exposure increases histologic markers of emphysema in HIV Tg rats, but not in littermate controls. Four to 5-month-old male HIV Tg rats and their littermate controls were exposed to CS for 2 months before sacrifice, removal of the lungs, fixations, and paraffin embedding. Tissues were then cut into 4-µm sections, deparaffinized, and rehydrated before H&E staining to assess mean linear intercept (L_m_). Cigarette smoke exposure did not alter the L_m_ of wild-type rats (A–B) but significantly increased the L_m_ of HIV Tg rats (C–D), suggesting the development of emphysema. All animals were males (n = 5–6). Individual values and means ± SEM. *P < 0.05 vs. groups at the end of individual lines.

**FIGURE 3.**
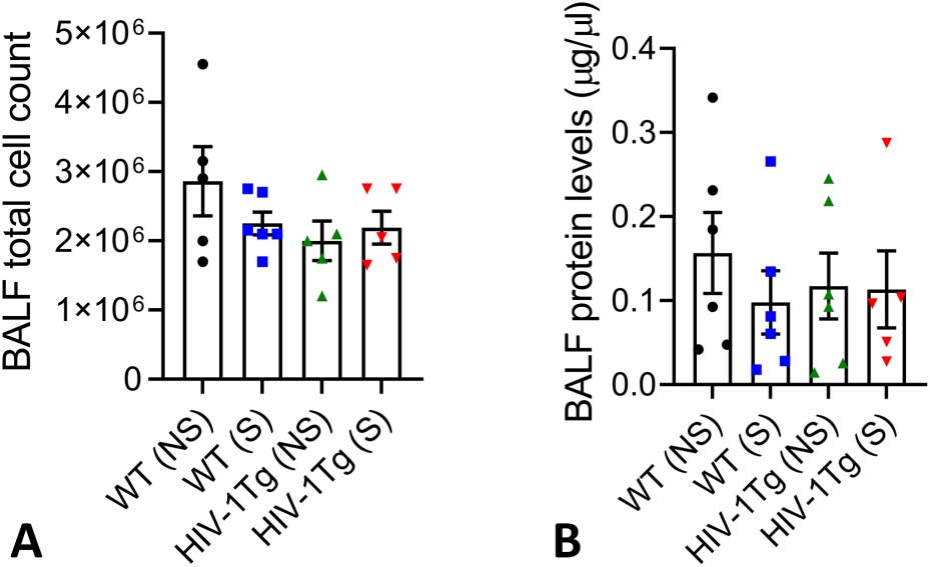
There is no significant difference total cell count or total protein levels in bronchoalveolar lavage fluid (BALF) of HIV Tg rats compared with littermate controls. Four to 5-month-old male HIV Tg rats and their littermate controls were exposed to CS for 8 weeks before collection of lavage fluid. A, Assessment of total cell count shows no significant difference between the HIV Tg rats and their littermates regardless of cigarette smoke exposure (NS = no smoke; S = smoke). B, Assessment of total protein via BCA protein assay shows no significant difference between the HIV Tg rats and their littermates regardless of cigarette smoke exposure. All animals were males (n = 5–6). Individual values and means ± SEM.

After exposing HIV transgenic rats and their littermate controls to cigarette smoke for 8 weeks, we assessed lung compliance and lung histology for evidence of emphysema. For the former measurement, we relied on the characteristic lung physiology of emphysema, in which lung recoil is decreased by loss of parenchymal elasticity resulting in a higher lung compliance (change in lung volume/change in lung pressure). As shown in Figure 1A and 1B, cigarette smoke did not alter the pressure–volume relationship or the compliance of wild-type rat lungs, suggesting that they did not develop significant emphysema after cigarette smoke exposure. By contrast, as shown in Figure 1C and 1D, the HIV transgenic rats had a significantly lower slope of the pressure volume loop and increased lung compliance, suggesting physiologically relevant emphysematous changes. After sacrificing the animals, we next examined their lung histology for confirmatory evidence of those changes. As shown in Figure 2, although air space appearances of HIV and wild-type rats were similar in the absence of cigarette smoke, the air spaces of wild-type rat lungs showed no evidence of dilation in response to cigarette smoke exposure, while those of the HIV transgenic rats showed a significant dilation of their air spaces (Fig. 2A). These changes in the gross appearance of the samples were accompanied by a significant increase in air space diameter as indicated by an increase in L_m_ in response to cigarette smoke exposure in the HIV transgenic rat lungs compared with their wild-type counterparts (Fig. 2B). Before sacrifice, bronchoalveolar lavage was performed for assessment of total cell count and total protein levels. As shown in Figure 3, there was no significant difference in BAL total cell count and protein levels of HIV Tg rats and their littermates, regardless of cigarette smoke exposure, indicating no significant difference in inflammatory cell recruitment or inflammation at the alveolar level.

### HIV Proteins Increase MMP-9 Levels and Activity in Alveolar Macrophages

**FIGURE 4.**
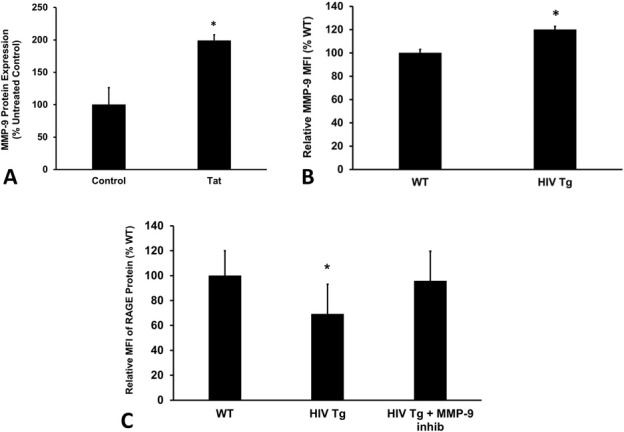
HIV increases matrix metalloproteinase-9 (MMP-9) levels and activity in rat alveolar macrophages. A, NR8383 cells were cultured with or without 10 ng/mL Tat for 48 hours. Western blot analysis was then performed using an MMP-9 antibody. Tat treatment significantly increased protein expression of MMP-9 compared with untreated control cells. *P < 0.05 vs. untreated control. B, Primary alveolar macrophages were obtained from male and female HIV transgenic rats and their littermate controls by whole-lung lavage and plated on a chamber slide. Cells were fixed and stained with an antibody to MMP-9 and counterstaining with a fluorescent secondary antibody. Data presented as mean ± SEM, n = 80–100 cells/group. * *P* < 0.05 compared with WT. C, Primary alveolar macrophages were again obtained from male and female HIV transgenic rats and their littermate controls by whole-lung lavage and plated on a chamber slide. Cells were treated ± direct MMP-9 inhibitor for 24 hours before fixing and staining with an antibody to the receptor for advanced glycation end products (RAGE) and counterstaining with a fluorescent secondary antibody. Data presented as mean ± SEM, n = 80–100 cells/group. *P < 0.05 vs. untreated control.

Given the known effects of MMP-9 in emphysema and prior data from human subjects suggesting elevated levels in the setting of HIV, we next treated a rat alveolar macrophage cell line (NR8383) with the HIV protein transactivator of transcription (Tat) to determine its effects on MMP-9. As shown in Figure 4A, MMP-9 protein expression, as assessed by Western blotting, increases 2-fold after 48 hours of Tat exposure (100% ± 26.4% vs 199% ± 9.13%, *P* < 0.05). We confirmed this finding in macrophages from the HIV transgenic rat (Fig. 4B), which also show a statistically significant increase in MMP-9 (100% ± 3% vs 120% ± 3%, *P* < 0.05). Next, to determine the biological activity of MMP-9, we made use of RAGE as an MMP-9 activity surrogate.^25^ Because RAGE is a key target of MMP-9, decreases in surface-level RAGE reflect increases in MMP-9 activity levels. As shown in Figure 4C, AM from HIV transgenic rats showed significantly lower levels of membrane-bound RAGE, as determined by cytoimmunofluorescence (Control: 100% ± 20%, HIV: 69% ± 24%, *P* < 0.05). Treatment with a direct MMP-9 inhibitor increased RAGE levels back up to the levels seen in wild-type AM, confirming that MMP-9 activity was responsible for the HIV-associated decrease in surface RAGE levels (HIV+MMP-9 inhibitor: 96% ± 24%).

### MMP-9 Levels are Significantly Increased in the Alveolar Macrophages and Plasma of HIV-1 Transgenic Rats Exposed to Cigarette Smoke

**FIGURE 5.**
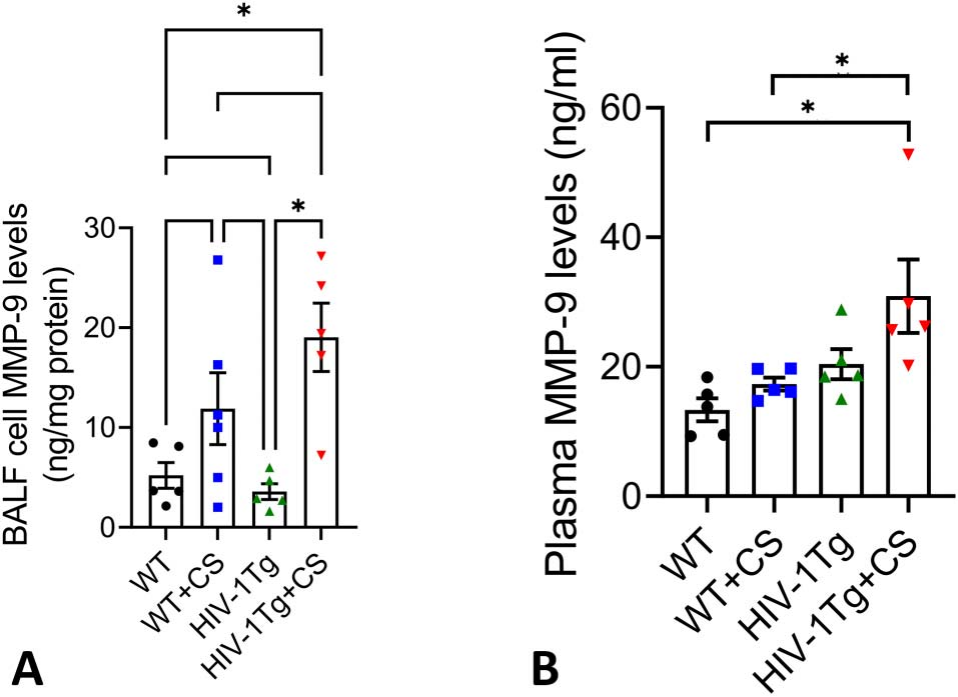
Cigarette smoke exposure increases alveolar macrophage and plasma MMP-9 levels in HIV-1 Tg rats. A, Four to 5-month-old male HIV Tg rats and their littermate controls were exposed to CS for 8 weeks before collection of lavage fluid and assessment of MMP-9 levels in alveolar macrophages and in circulating plasma by ELISA. Although cigarette smoke increased levels of alveolar macrophage MMP-9 in wild-type rats, the difference was statistically insignificant. B, In HIV transgenic rats exposed to cigarette smoke, levels were significantly higher than in their unexposed counterparts (and significantly higher than both WT groups). Similarly, plasma MMP-9 levels were significantly higher in HIV-1 Tg than the WT rats after exposure to cigarette smoke. All animals were males (n = 5–6). Individual values and means ± SEM. *P < 0.05 vs. groups at the end of individual lines; one-way ANOVA followed by Tukey post hoc testing.

HIV-1 transgenic male rats and their male littermate controls were exposed to cigarette smoke for 8-weeks and then sacrificed to obtain alveolar macrophages through bronchoalveolar lavage and plasma from blood. As shown in Figure 5, MMP-9 protein levels were significantly elevated in the (Fig. 5A) alveolar macrophage (11.89 ng/mg protein in wild type + CS vs. 19.04 ng/mg protein in HIV-1 Tg + CS, *P* < 0.05) and (Fig. 5B) plasma (17.33 ng/mL in wild type + CS vs 30.91 ng/mL in HIV Tg rats + CS) of HIV-1 transgenic rats exposed to cigarette smoke compared with their littermate controls.

### Circulating MMP-9 and RAGE Levels are Increased in Human Smokers With HIV

**FIGURE 6.**
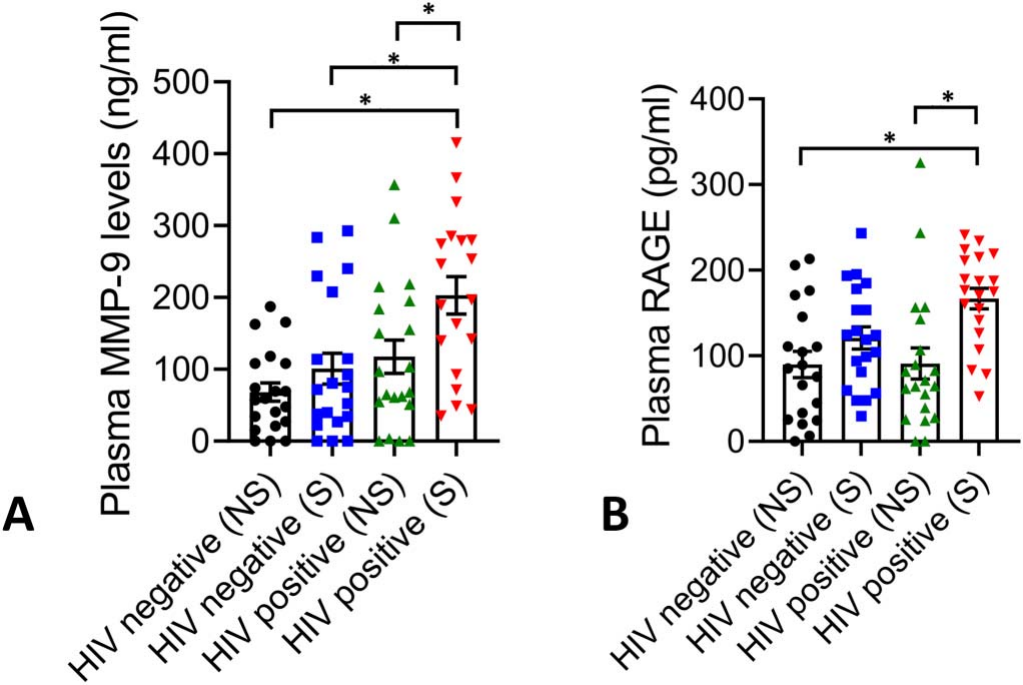
Plasma MMP-9 and RAGE levels are higher in the plasma of human subjects with tobacco dependence and HIV. MMP-9 and RAGE levels were measured by ELISA in the plasma of subjects with (S) and without (NS) tobacco dependence and with and without HIV. A, Those without tobacco dependence or HIV had significantly lower levels of plasma MMP-9 than those with HIV and tobacco dependence. B, Those without tobacco dependence had significantly lower levels of plasma RAGE than those with HIV and tobacco dependence, correlating with MMP-9 expression. *P < 0.05 vs. groups at the end of individual lines.

To confirm the clinical relevance of this pathway, we then assessed plasma MMP-9 in human subjects with and without HIV. As shown in Figure 6A, MMP-9 levels, as assessed by ELISA, were significantly higher in smokers with HIV (202.9 ng/mL) than in their nonsmoking counterparts (117.5 ng/mL) and in smokers and nonsmokers without HIV (100.8 ng/mL and 68.3 ng/mL, respectively). Figure 6B shows that the levels of soluble RAGE, as assessed by ELISA, correlate with these plasma MMP-9 levels and was significantly elevated in smokers with HIV.

## DISCUSSION

Growing recognition of the chronic comorbidities that accompany HIV infection has brought with it renewed attention to the mechanisms by which those complications occur. In this study, we provide novel evidence that smoking increases susceptibility to HIV-associated emphysema and implicate the HIV-1 viral protein Tat and the host protein MMP-9 as key actors in the process. More broadly, we believe this study connects to our prior work demonstrating that HIV increases oxidative stress in the alveolar space and that oxidative stress increases MMP-9 activity to provide a compelling overarching narrative of HIV-associated emphysema. Specifically, by decreasing antioxidant defenses, HIV proteins promote MMP-9 activity, which could subsequently prime the lung for the development of emphysema.

To assess the relationship between HIV, MMP-9, and emphysema, we first made use of the HIV transgenic rat model to determine whether HIV proteins are sufficient to increase the risk for emphysema. We have previously found this model to be a useful surrogate for human biology in pathways related to alveolar macrophage function.^16,24^ As shown in Figures 1 and 2, the results of both physiologic and histologic analyses convincingly establish that exposure to cigarette smoke for 8 weeks induces an emphysematous lung phenotype in HIV-1 transgenic rats but not in WT rats. These emphysematous changes did not correlate with an overall increase in alveolar inflammation as shown by the lack of difference in total cell count and total protein level in the BAL of HIV Tg rats and their littermates (Fig. 3). In addition, we note that our finding of increased MMP-9 activity in the rat lung accords well with previous studies performed in human subjects,^26^ as well as our data from rat and human plasma (Figs. 5B and 6A). Notably, the experiments involving cigarette exposure only included male rats because of a plethora in the litter and in an effort to minimize in vivo variation. This is a limitation of our study, however our experiments using this rat model without cigarette exposure used both male and female rats (Fig. 4B and 4C) and did not show a sex-based variability in MMP-9 expression. Although there is a previous work noting sex-related differences in matrix metalloproteinase expression, it is predominantly in cardiovascular and neurologic disease states.^27,28^ However, as comparisons of our cigarette-exposed rats included only male groups, we still consider their relative differences to be significant and noteworthy.

After establishing a clear role for HIV in driving smoking-related emphysema, we next wanted to determine whether HIV increases MMP-9 in the alveolar macrophage. Although there are many cells in the alveolar space that likely contribute to the development of emphysema, in its role as the resident innate immune effector of the lung, the AM is uniquely positioned to regulate a variety of innate immune effector and homeostatic functions. We therefore began our studies with a focus on the macrophage, specifically with an in vitro model of HIV protein exposure (Tat) that we based on concentrations of the protein found in the alveolar space which was previously found to decrease antioxidant defenses.^29^ As shown in Figure 4A, exposure to Tat protein indeed increases MMP-9 protein expression. We next assessed the level of MMP-9 present in the HIV transgenic rat model and found that MMP-9 was increased significantly when compared with wild-type littermate controls (Fig. 4B). Given the significant change in MMP-9 levels, we sought to determine whether MMP-9 activity was significantly different as well. We therefore made use of RAGE as a surrogate for MMP-9 activity. We previously determined that levels of membrane-bound RAGE vary inversely with MMP-9 activity, given its status as a target for cleavage by MMP-9.^25^ Here, we made use of a direct MMP-9 inhibitor to corroborate our previous findings and confirm that this assay serves as an appropriate correlate of MMP-9 activity (Fig. 4C). In a previous study, we determined that an excess of soluble RAGE (sRAGE, or RAGE that has been cleaved from the cell membrane) acts as a dead-end for inflammatory signals in the alveolar space, binding ligands without allowing their normal processing and propagation.^25^ The function of the excess sRAGE in HIV has yet to be determined but may present a further interesting avenue for investigation of HIV-induced innate immune deficiency.

Finally, we sought to determine the relevance of the MMP-9 pathway in HIV by assessing its levels in human subjects with HIV. Previous studies of MMP-9 have shown mixed results. One recent study found that MMP-9 levels are elevated in the lungs of people with HIV,^30^ but another found that MMP-9 is decreased in their serum.^31^ In this context, our findings of higher MMP-9 levels in plasma of smokers with HIV provide important clinical context for the pathway under investigation. The reason people with HIV develop more robust increases in MMP-9 in the context of smoking is unclear, but we would hypothesize that smoking-induced increases in oxidative stress are unable to elicit the protective Nrf2 responses they do in subjects without HIV because of their baseline impairments in Nrf2 activity.^16^ We also demonstrated that plasma levels of MMP-9 are similar at baseline in people with and without HIV, but that smoking increases these levels only in people with HIV, further supporting the clinical relevance of this pathway to human subjects with HIV-associated emphysema (Fig. 6). These results also suggest a role for plasma MMP-9 levels as a potential biomarker for HIV-associated emphysema. Future studies, including lung function assessments, are needed to correlate this to physiologic data.

In summary, in determining a role for MMP-9 and antioxidant defenses in HIV-associated emphysema, we believe these data set the stage for future studies of host-directed therapies, particularly therapies designed to attenuate HIV-associated oxidative stress to diminish MMP-9 activity. In addition, we plan to investigate emphysema outcomes in the setting of MMP-9 inhibition in this HIV rat model to cement the role of MMP-9 in the disease pathway. As in the case of HIV-associated pulmonary hypertension,^32^ our data support the emerging hypothesis that unique mechanisms are responsible for lung complications in people with HIV. We believe that uncovering these unique mechanisms will allow us to better tailor our therapeutic approaches to this vulnerable population.
